# Linguistic drivers of misinformation diffusion on social media during the COVID-19 pandemic

**DOI:** 10.1007/s43039-021-00026-9

**Published:** 2021-05-29

**Authors:** Giandomenico Di Domenico, Annamaria Tuan, Marco Visentin

**Affiliations:** 1 Faculty of Business and Law, University of Portsmouth, Richmond Building, Portsmouth, PO1 3DE UK; 2 Department of Management, University of Bologna, Via Capo di Lucca 34, Bologna, Italy

**Keywords:** Covid-19, Misinformation, Twitter, Linguistic analysis, Machine learning

## Abstract

In the wake of the COVID-19 pandemic, unprecedent amounts of fake news and hoax spread on social media. In particular, conspiracy theories argued on the effect of specific new technologies like 5G and misinformation tarnished the reputation of brands like Huawei. Language plays a crucial role in understanding the motivational determinants of social media users in sharing misinformation, as people extract meaning from information based on their discursive resources and their skillset. In this paper, we analyze textual and non-textual cues from a panel of 4923 tweets containing the hashtags #5G and #Huawei during the first week of May 2020, when several countries were still adopting lockdown measures, to determine whether or not a tweet is retweeted and, if so, how much it is retweeted. Overall, through traditional logistic regression and machine learning, we found different effects of the textual and non-textual cues on the retweeting of a tweet and on its ability to accumulate retweets. In particular, the presence of misinformation plays an interesting role in spreading the tweet on the network. More importantly, the relative influence of the cues suggests that Twitter users actually read a tweet but not necessarily they understand or critically evaluate it before deciding to share it on the social media platform.

## Introduction

The COVID-19 pandemic has proven to be not only an epidemic but also an “infodemic” given that the spreading of the virus has been accompanied by an explosion of fake news and, in general, misinformation about the disease (WHO, 2020). Previous research has shown how misinformation travels faster and farther than genuine information on social media (Vosoughi et al., 2018). Unfortunately, this trend has been confirmed also during the COVID-19 outbreak, as the amount of information shared from untrustable and genuine sources was comparable (Yang et al., 2020). Indeed, during the last months, we have witnessed a massive diffusion of misinformation regarding the virus, mainly in the form of conspiracy theories about it being created as a biological weapon in China and transmitted through the 5G network (Ahmed et al., 2020; Di Domenico & Visentin, 2020). Particularly, the 5G conspiracy has spread massively on social media, becoming a trending topic on Twitter and arousing anger among users (Jolley & Paterson, 2020). However, misinformation does not only spread on Twitter but it might overcome the limits of the platform, causing tangible harm. For instance, after the spread of 5G misinformation on Twitter, several cell phone masts have been vandalised in the United Kingdom (BBC, 2020; Brewis, 2020). As a consequence, these events affected companies that have been investing in the development of the 5G network, in particular Chinese technology companies such as Huawei, tarnishing their reputation (Di Domenico & Visentin, 2020).

From a marketing perspective, it becomes crucial to see how companies could understand and prevent the spread of misinformation before it might damage their reputation.

Together with social media algorithmic characteristics (Di Domenico et al., 2021), the language that social media users adopt to communicate meanings (Hewett et al., 2016) can be a factor in fostering the spreading of misinformation. As people extract meaning from information based on their discursive resources and their skillset (see Marwick, 2018), language is crucial in the evolution of sharing misinformation*.*

On Twitter specifically, the choice of the words used and the hashtags selected enables or restricts the possibility of spreading contents within *echo chambers*, digital environments where misinformation can thrive (Hewett et al., 2016; Quattrociocchi et al., 2016). Twitter is one of the most used social media platforms with 187 million monetizable daily active users worldwide (Statista, 2021). A growing body of literature investigates Twitter from the consumer point of view (e.g. Hennig-Thurau et al., 2015; Kim & Song, 2016) and only recently the influence of the linguistic style of tweets on retweets rates has been more deeply investigated (e.g. Aleti et al., 2019; Labrecque et al. 2020). The analysis of Twitter user-generated textual data (e.g. statuses and tweets) can provide unprecedented possibilities to analyse how individuals use language to communicate misinformation on social media and boost contents’ visibility. However, marketing literature about misinformation lacks empirical studies on the effects of the content and linguistic style of social media user-generated contents on the spreading of misinformation.

To fill in this gap, we propose an analysis of a set of 4923 tweets containing the hashtags #5G and #Huawei to investigate the effect of textual features (namely: *linguistic style, certain language, text complexity, misinformation*) and users’s profile characteristics on the probability of a tweet to be retweeted and on the intensity of retweets. We analysed psycholinguistic cues extracted using Linguistic Inquiry and Word Count (LIWC), which is considered as a transparent text analysis program that counts words in psychologically meaningful categories (Tausczik & Pennebaker, 2010). Moreover, we complement our analysis by using also non-textual cues including users’ status on Twitter and the inclusion of media elements in the tweet. We then applied a machine learning (ML) approach (*gradientBoost*, Chen & He, 2020; Natekin & Knoll, 2013) to investigate the pattern of textual and non-textual characteristics of a tweet in determining users’ response in terms of retweeting behavior. Finally, we compared the predictive ability of the ML approach to a traditional logistic regression, finding the superiority of the former in reducing false positives and false negatives.

## Fake news and misinformation

Misinformation (e.g. fake news, hoaxes, propaganda and conspiracy theories) are nothing new (Tandoc et al., 2018), but they have found in social media a powerful medium to spread broadly and magnify their reach. Indeed, previous research has found that misinformation and fake news travel faster and farther on social media platforms (Vosoughi et al., 2018). This is due to various reasons (Di Domenico et al., 2021): first, social media have disintermediated the access to production and consumption of news (Lazer et al., 2018) allowing everyone to produce news (Allcott & Gentzkow, 2017). Second, social media algorithms foster the creation of so-called *echo* chambers, namely groups of like-minded people who acquire information from a limited set of sources (Del Vicario et al., 2019) reinforcing their pre-existing beliefs (Schmidt et al., 2018; Unkelbach et al., 2019). Finally, while social media were born as platforms to connect people from all over the world, they are being adopted as the primary source of information (Newman et al., 2017; Schmidt et al., 2017).

Fake news represents a form of “digital pollution” that makes the environment hard for marketers to navigate (Fulgoni & Lipsman, 2017). Fake news is defined as news “intentionally and verifiably false and could mislead readers” (Allcott & Gentzkow, 2017, p. 213). It is represented as “fabricated stories”, intentionally false but realistic, which appeal to users’ previous beliefs (e.g.; Di Domenico & Visentin, 2020; Talwar et al., 2020; Tandoc et al., 2018; Visentin et al., 2019). To date, most of the literature on misinformation has tried to understand the drivers underpinning fake news belief and sharing on social media. One of the most important drivers of belief in fake news was found to be confirmation bias (Quattrociocchi et al., 2016), that is *“the human tendency to acquire information adhering to one’s system of beliefs”* (Del Vicario et al., 2019, 10:2). Confirmation bias explains the human tendency to select information consistently with prior beliefs, thus reinforcing them (Lewandowsky et al., 2012). Cognitive abilities also play an important role in fostering individuals’ belief in misinformation (Pennycook et al., 2018). Specifically, less analytic individuals present greater propensity to endorse suspect beliefs such as paranormal and superstitious beliefs (Pennycook et al. 2012), conspiracy beliefs (Swami et al. 2014), and pseudo-profound bullshit (Pennycook et al. 2015) which, in turn, lead to belief in fake news. Moreover, delusion prone individuals are more likely to believe in fake news (Bronstein et al. 2019). Consequently, people who were more willing to think analytically are less likely to believe articles including misinformation regardless of their partisan alignment (Pennycook and Rand, 2019). Finally, emotions also can prompt belief in fake news (Martel et al., 2020).

Moreover, scholars have also analysed the patterns of misinformation and fake news spreading on social media finding that misleading contents are mostly generated by ordinary users (Jang et al., 2018) who continuously modify and change text characteristics to “repackage” fake news giving it new popularity (Shin et al., 2018).

By mainly focusing on the psychological drivers of belief in and sharing of misinformation, existing scholarship on this intriguing topic leaves some gaps in the understanding of the textual characteristics that make misinformation achieve virality. As individuals create and extract meanings from texts (Marwick, 2018), the analysis of language is crucial in the comprehension of fake news and misinformation sharing behaviours. Moreover, the use of specific words or hashtags can determine the overall reach of the content on social media, stimulating the reverberating system of the “*echoverse*” (Hewett et al., 2016). For this study, we focus on Twitter for two reasons. Firstly, the spreading of misinformation through this platform is pervasive and it has been chosen as the context of several misinformation studies (e.g. Vosoughi et al., 2018). Secondly, the text of tweets constitutes a valuable starting point to study how language is built (Berger et al., 2020) and affect others (by the means of retweets).

In our analysis, we focus on textual (linguistic style, level of certainty, complexity and misinformation content) and non-textual (user’s characteristics and inclusion of media) cues of tweets in their effect on (1) the probability of the tweet being retweeted and (2) the virality of tweets, in terms of the total number of retweets. Consequently, we formulate the following research questions for this study:

- RQ1: Do textual and non-textual characteristics of tweets cue virality, i.e. retweet?
- RQ2: Do textual and non-textual characteristics of tweets increase virality, i.e. the number of retweets?

## Textual and non-textual cue stimulating retweets

In order to answer our research questions, we draw upon cueing theory, which suggests that visual and verbal stimuli affect different levels of individual responses (e.g. Labrecque et al. 2020; Olson & Jacoby, 1972; Wedel & Pieters, 2014; Wu et al., 2016; Visentin & Tuan, 2020).

In the context of Twitter, cues are represented by the words used (e.g. content words and function words), the media included (e.g. images, urls) and the type of user profile (e.g. number of followers). These cues affect consumers’ sharing behavior in terms of retweets. In particular, in this paper we focus on textual cues represented by the *linguistic style* of the text, the level of *certainty* of the statements, the *complexity* of the text and the presence of *misinformation*. We complement our framework by considering also non-textual cues such as the *status of the user* and the *use of media*.

**Fig. 1:**
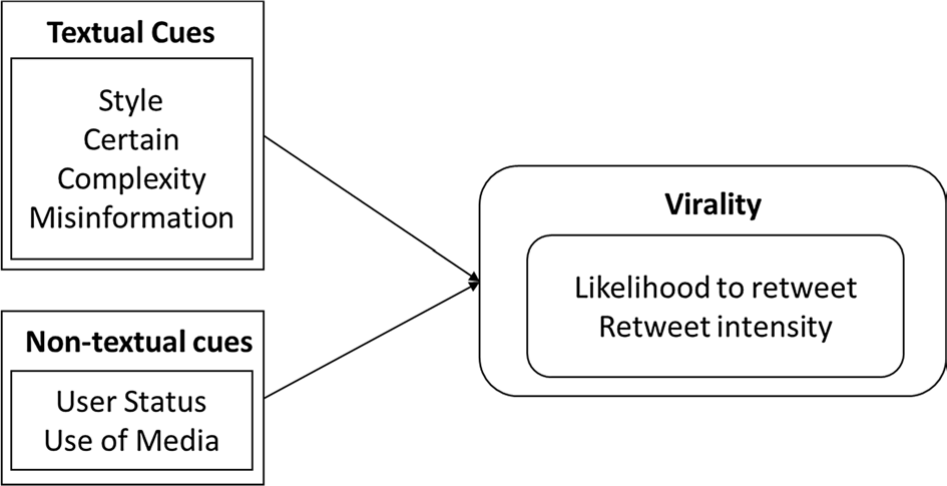
Conceptual framework

Figure 1 depicts the theoretical framework of our analysis.

### Textual cues

Regarding the words used in tweets, the marketing literature has traditionally focused on the content of social media posts, mainly from a company perspective (e.g. Ashley & Tuten, 2015; de Vries et al., 2012). However, in recent years the attention has moved towards the *linguistic style* used in the text suggesting that content words and function words (e.g. adverbs, pronouns) are equally important to communicate meaning, to develop mental imagery, to direct the attention of the reader (e.g. Aleti et al, 2019; Douglas et al., 2017) as well as to affect engagement (e.g. retweets) (e.g. Cruz et al. 2017; Labrecque et al., 2020). By looking at subtle and often subconscious choices about linguistic style elements, we may indeed gain useful insights about how users construe the world around them.

Aleti et al. (2019) analyzed, for instance, the narrative/analytical, internally/externally focused (*clout)*, and negative/positive emotional styles (*tone*) in tweets by celebrities and their effect on word of mouth. Berger et al. (2020), by analyzing social media posts created and shared during live political debates, found that tweets containing a more analytical language—but not authentic language style—were more likely to be shared after the debate. Humphreys et al. (2020) found that consumers use more abstract (concrete) language in their online search during the first (last) stages of the shopping journey. Akpinar and Berger (2017) also suggest that highly arousing text (*tone*), both highly positive and highly negative, is more likely to be shared frequently and widely since the audience is more receptive to a message when it arouses affective states.

Moreover, also text *complexity* and the presence of *certainty in language* in the statements play a crucial role in affecting sharing behaviors. *Complexity* refers to the presence in texts of a high *number of words*, *propositions*, *long* words and more words related to *cognitive mechanisms* (Tausczik & Pennebaker, 2010; Zhou et al., 2004). In the case of misinformation, text *complexity* may be reduced because of the cognitive load required to maintain a story that is contrary to experience. Literature is still scant in this regard but we expect that the more a text is complex, the less it will be convincing and that, in turn, this text will be shared less. *Certainty* refers to a sense of conviction or confidence that characterizes language. Previous research suggests that certain language increases consumer engagement to brands’ social media messages (Pezzuti et al., 2021; Leek et al., 2019). The same happens for individuals who appear to be more powerful by using words that denote certainty (Hart & Childers, 2004).

Furthermore, also the presence of *misinformation* in the content of the text may cue social media users’ behavioral response. As previously stated, misinformation has found a fertile ground on social media, and in particular on Twitter, allowing people to disseminate unreliable information and misleading content (Di Domenico et al., 2021; Del Vicario et al., 2019). However, when this content aligns with an individual’s perception of the world, the possibility of being shared increases irrespective of factual truthfulness and objective reality, affecting therefore the virality of the tweet.

### Non-textual cues

In addition to the content and the style of the tweet, we also consider cues that are peripheral to the text, like the *user status* and the *use of media*. First, previous literature suggests that the number of followers, the number of friends and the volume of statuses act as an indicator of source influence for the reader (Xu & Zhang, 2018). These indicators shape the *user status* and represent cues which capture the attention of the user, providing information about the level of authority of the user profile. Second, the inclusion of images or URLs characterizes the levels of vividness and interactivity of the text, which ultimately influence virality (De Vries Gensler & Leeflang, 2012; Tellis et al. 2019). These indicators are non-textual cues of a tweet represented by the *use of media*.

## Empirical analysis

In this study, we focus on the diffusion of misinformation regarding the company Huawei being associated with false rumours regarding insidious connections between the spread of coronavirus and the deployment of the 5G network. To this aim, we scraped from Twitter 4923 tweets containing the hashtags #5 g #Huawei on May, 8 2020. The dataset was streamlined by removing verified users and retweets. The refined dataset includes 1103 tweets.

First, we manually coded tweets to scrutinize whether they were related to *misinformation* or not. The three authors manually coded the content of tweets, founding that 223 out of 1103 tweets were misinformative, reaching a near-perfect degree of agreement (Krippendorff α = 0.971). These are some examples of tweets coded as misinformation:

"This Genocide facilitated by HUAWEI’s digital surveillance of Uyghurs. UK must pull out of HUAWEI 5G deal.”

“@Huawei Don't need 5G from a country that let covid 19 out. So no thanks”

“@CDORF4REALRED @darrenstanton @Huawei Lets finish with the Corona pandemic first, forcing our selves into another problem.5G is evil.”

Second, we performed the automated text analysis of tweets by using LIWC (Linguistic Inquiry and Word Count; Tausczik & Pennebaker, 2010), software commonly used to detect meaning from the text and widely adopted in the marketing literature (e.g. Berger et al., 2020). Third, we empirically assessed the effect of the linguistic cues of the tweet on the capability of a tweet to be retweeted. Fourth, we tested the predictive ability of the model.

In detail, we used the summary variables provided by LIWC to account for *analytical language, authentic language, clout* and *tone*. The summary variables we used are algorithms included in the LIWC software program which previous linguistic research has thoroughly validated internally and externally (Aleti et al., 2017; Pennebaker et al. 2015). They have already been used successfully in a variety of marketing and management studies (Akpinar et al. 2018; Hwong et al. 2017; Margolin & Markowitz, 2018; Tausczik & Pennebaker, 2010). These variables range from 0 to 100. The summary variable *analytic* captures the narrative/analytical style. In particular, the presence of more exclusive words (e.g. but, while, whereas), more self-and-other references, and less negative emotion denotes an *analytical language* whereas a *narrative language* is characterized by the presence of more adverbs, auxiliary verbs, conjunctions, negations. The variable *authentic* combines the positive loading of first‐ and third‐person singular pronouns, third‐person plural pronouns, and exclusive words (e.g., but, except, and without) with the negative loading of negative emotions and motion verbs (e.g., arrive, drive, and go). Conversely, low scores on this variable relate to more distance from the self and a more deceptive language (Barrett et al., 2002; Newman et al., 2003). *Clout* refers to the degree to which texts contain an internally or externally focused style (Pennebaker et al. 2015). *Tone* captures the negative (e.g., hurt, nasty, ugly) and positive (e.g., love, nice, sweet) emotional style.

To account for *text complexity*, we included the categories of LIWC which refer to a complex language: word count (*Word Count*), presence of prepositions (*Prepositions*), words with more than six letters (*Long words*) and cognitive mechanisms (*Cognitive Mecs*). To account for *certainty in language*, we included words communicating possibility (*Tentativeness*) and certainty (*Certainty*). Table 1 provides descriptive statistics.

**Table 1:** Descriptive statistics

	Fake/Nofake	Mean	Std. Deviation
Analytic	0	82.05	24.88
	1	69.69	28.02
Clout	0	63.82	25.01
	1	65.28	26.36
Authentic	0	14.95	23.14
	1	15.90	23.79
Tone	0	36.88	31.11
	1	33.99	32.56
Word count	0	27.89	12.73
	1	30.08	14.35
Long words	0	18.02	9.24
	1	14.93	9.01
Prepositions	0	11.88	5.74
	1	10.90	5.87
Cognitive mecs	0	7.40	5.65
	1	8.42	6.85
Tentativeness	0	1.14	2.34
	1	1.48	2.42
Certainty	0	0.63	1.84
	1	1.04	3.72

In order to answer to our first research question and to find which tweets’ cues affect the possibility of a tweet to be retweeted, we estimated a Logit model on the retweet count of the 1103 tweets. We used the dummy variables *Retweet/No retweet* as the dependent variable, *Analytic, Authentic, Clout, Tone*, *complex language, certain language* and *misinformation* as independent variables. We also added the dummy variable *misinformation*, the user’s status on Twitter and the presence of media elements as controls. Models 1–6 provide partial model estimates, Model 7 provides the full model. In particular, we first calculated the intercept model, taken as a base model for further comparisons (Model 1). Then, we calculated Model 2 using the *linguistic style* variables (*Analytic, Authentic, Clout, Tone)*, Model 3 using *certain language* variables (*Tentativeness* and *Certainty*)*,* Model 4 using *complex language* variables (*Word Count, Prepositions, Long Words, Cognitive Mecs*), Model 5 using *Misinformation* and Model 6 including the non-textual cues (*Followers Count, Friends Count, Statuses Count, Urls, Media*). Table 2 reports the results of the Logit analysis using standardized variables.

**Table 2:** Logit analysis

	Dependent variable: retweet yes/no						
	(1)	(2)	(3)	(4)	(5)	(6)	(7)
Linguistic style							
Analytic		0.431*** (0.110)					0.140 (0.149)
Authentic		0.057 (0.091)					0.095 (0.103)
Clout		0.003 (0.092)					−0.054 (0.103)
Tone		0.124 (0.083)					0.117 (0.087)
Certainty in language							
Tentativeness			−0.103 (0.091)				0.024 (0.110)
Certainty			−0.024 (0.089)				0.145 (0.108)
Complexity							
Word Count				0.321*** (0.087)			0.311*** (0.094)
Prepositions				0.181** (0.088)			0.137 (0.109)
Long Words				0.244*** (0.088)			0.216** (0.095)
Cognitive Mecs				−0.294*** (0.103)			−0.261** (0.132)
Misinformation							
Misinformation					0.001 (0.202)		0.222 (0.220)
Non-textual cues							
Followers count						0.146* (0.087)	0.125 (0.082)
Friends count						0.245 (0.189)	0.202 (0.180)
Statuses count						−0.088 (0.099)	−0.071 (0.098)
Urls						0.507*** (0.180)	0.501** (0.213)
Media						0.424* (0.232)	0.186 (0.251)
Intercept	−1.668*** (0.082)	−1.725*** (0.087)	−1.672*** (0.083)	−1.768*** (0.090)	−1.669*** (0.093)	−2.047*** (0.147)	−2.168*** (0.180)
Observations	1103	1103	1103	1103	1103	1103	1103
Log Likelihood	−482.494	−472.567	−481.740	−463.192	−482.494	−469.134	−450.910
Akaike Inf. Crit	966.989	955.134	969.480	936.385	968.989	950.267	935.819

*p < 0.1; **p < 0.05; ***p < 0.01

Then, to answer to our second research question and to account for the effects of the tweets’ cues to affect the virality of the tweet, we considered the subset of tweets in our database that have been retweeted. Thus, we estimated a Poisson model on the retweet count for the 175 retweets in our dataset. We accounted for the same independent variables of the previous model but we used the *retweet count* as the dependent variable. Also in this case, Models 1–6 provide partial model estimates, Model 7 provides the full model. Table 3 reports the results of the Poisson analysis using standardized variables.

**Table 3:** Poisson analysis

	Dependent variable: retweet count						
	(1)	(2)	(3)	(4)	(5)	(6)	(7)
Linguistic style							
Analytic		0.083** (0.036)					0.109** (0.051)
Authentic		0.111*** (0.034)					0.105*** (0.038)
Clout		0.318*** (0.039)					0.363*** (0.042)
Tone		−0.152*** (0.036)					−0.104*** (0.040)
Certainty in language							
Tentativeness			−0.074* (0.038)				0.002 (0.057)
Certainty			−0.018 (0.035)				0.073 (0.045)
Complexity							
Word count				0.386*** (0.033)			0.276*** (0.036)
Prepositions				0.088** (0.036)			0.013 (0.044)
Long words				−0.058 (0.036)			0.103*** (0.039)
Cognitive mecs				−0.176*** (0.039)			−0.102* (0.052)
Misinformation							
Misinformation					0.918*** (0.069)		0.825*** (0.081)
Non-textual cues							
Followers count						0.592*** (0.032)	0.539*** (0.039)
Friends count						−0.488*** (0.044)	−0.491*** (0.048)
Statuses count						0.075** (0.032)	0.228*** (0.034)
Urls						−0.316*** (0.077)	−0.106 (0.084)
Media						−0.061 (0.091)	−0.275*** (0.099)
Intercept	1.603*** (0.034)	1.552*** (0.036)	1.600*** (0.034)	1.512*** (0.037)	1.327*** (0.044)	1.727*** (0.063)	1.248*** (0.076)
Observations	175	175	175	175	175	175	175
Log likelihood	−1147.204	−1105.223	−1145.049	−1067.517	−1066.798	−1018.979	−857.673
Akaike Inf. Crit	2296.409	2220.447	2296.097	2145.034	2137.595	2049.958	1749.346

*p < 0.1; **p < 0.05; ***p < 0.01

### Results

The results of the Logit analysis on the possibility of a tweet to be retweeted are displayed in Table 2. All the models, except Model 3 using *certain language* variables and Model 5 using *misinformation* are significantly different from the null model (P(χ^2^, df) < 1e−03). The differences between the full model and all the sub-models are significant (P(χ^2^, df) < 1e−03). Moreover, the intercepts are significant and negative in all models.

In Model 7, we find a significant effect only of the variables related to the complexity of the language, namely the word count (*Word Count*), words with more than six letters (*Long Words*) and cognitive mechanisms (*Cognitive Mecs*), suggesting that longer and more complex tweets are retweeted more. It is worth noting that the variable *Misinformation* does not report a significant value meaning that the retweet does not depend on the veracity of the tweet but rather on other textual and non-textual cues. Finally, we also find a significant and positive effect of the presence of URLs suggesting that when the tweet is complemented by a link to external sources it catches more attention by the reader, eliciting retweeting behaviors. These results provide interesting insights about the cues that lead to a higher likelihood of a tweet being retweeted, answering our first research question. Specifically, we found that the presence of complex language and links to external sources in tweets positively affect the probability of a tweet being retweeted.

A different picture is provided by modelling the ability of a tweet to accumulate retweets. In fact, by considering only the tweets which have been retweeted, the Poisson model allows us to answer to the second research question, i.e. *which are the main tweets’cues that increase the virality of tweets* (Table 3). The differences between the full model and all the sub-models are significant (P(χ^2^, df) < 1e-03). In this case, all the models are significantly different from the null model (P(χ^2^, df) < 1e-03). Moreover, the intercepts are significant in all models.

In the full Model 7, we find a positive and significant effect of *Analytic, Authentic, Clout* and a negative effect of *Tone.* These results suggest that the linguistic style of the tweet affects the probability of the tweet to be retweeted. In particular, tweets are more retweeted when they contain a more analytical, authentic and confident language. Whereas negative tweets are less appreciated by the users. Even in this case, the significant effect of the *complexity* variable is maintained. Noteworthy, the presence of *Misinformation* is significant in the full Model 7 and partial Model 5. Interestingly, in this case, also the non-textual cues play an important role in determining the virality of tweets. In particular, the *Followers Count* and the volume of tweets (namely, *Statuses Count*) have a positive and significant effect on the *retweet count*. *Friends count* and the presence of images (namely, *Media*) have a negative effect, instead.

### Predictive analysis

Finally, to provide additional managerial relevance to this analysis, we performed a comparison between the predictive ability of a traditional logit model and a machine learning boosting machine (e.g.: Friedman, 2001, 2002; Natekin & Knoll, 2013). In particular, we analyzed the forecasting performance of the models to predict a retweet based on the characteristics of the tweet.

We compared the logit approach, widely used in management studies, to a machine learning (ML) approach based on pattern recognition (Freund & Schapire, 1997; Friedman, 2002) since the language descriptors of a text provide a pattern. In particular, among the plethora of pattern recognition engines available in the ML tradition, we selected a *gradient boosting* algorithm (Chen & He, 2020; Friedman, 2001, 2002; Natekin & Knoll, 2013; Qian et al., 2020; Ridgeway, 1999; Ridgeway & Ridgeway, 2004). The *gradient boosting* approach is based on a combination of Classification And Regression Trees (CART; Friedman, 2001; Friedman et al., 2000; Ridgeway, 1999; Therneau & Atkinson, 1997) as base classifiers, each obtained on a bootstrap replicate of the training set (e.g.: Friedman, 2002). First, CART models split each node (i.e. a group of observations) based on the predictor that ensures the best reduction of variance. Second, bootstrap replicates are copies of the original training set generated by randomly selecting observations with re-immissions in order to preserve the original sample size. Third, following Friedman’s *GradientBoosting* machine (Friedman, 2001, 2002), the algorithm iteratively updates on the previous classification to reduce the under-fitted predictions.

This approach provides data sets that over-represent some observations and other data sets that under-represent them. Ultimately, CARTs on each bootstrap replicates will follow this over(under)-representation of patterns providing a low-performing family of classifiers. Nevertheless, their combination will provide a high-performing forecasting tool (Chen & He, 2020; Friedman et al., 2000; Friedman, 2001, 2002; Natekin & Knoll, 2013; Qian et al., 2020; Ridgeway, 1999; Ridgeway & Ridgeway, 2004). Noteworthy, current implementations of *gradient boosting* machine provide a relative ranking of the variables (*predictors* in the ML tradition) that can be operationally used.

To perform the comparison, we randomly selected 80% of classified tweets and used as training set, i.e. the data set used to estimate the models (logit and *gradient boosting*). We then used the remaining 20% as the test set.

**Fig. 2:**
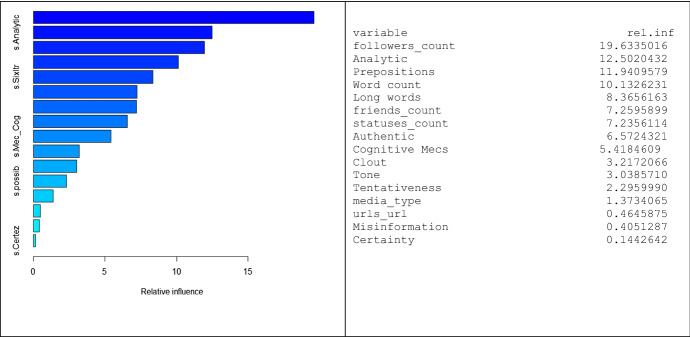
Relative influence of variables

Figure 2 reports graphically and numerically the relative influence of the variables used by the *gradient boosting* algorithm. In particular, by weighting the basic tree classifiers (as described in Friedman, 2001), the graphical interface orders the variables consistent to their relative influence in the final classification model. Results show that the more important variables used are related to the *analytic language, complex language* and status, with a prominent role of *followers, friends* and *statutes* counts. Conversely, *media* and *misinformation* are marginally relevant. Overall, these results reveal that people mostly rely on the user’s *status* and on the *text complexity* to decide whether or not to retweet. In this line, it appears that a tweet is retweeted or not depending on the fact that it is read but not understood.

Finally, the *gradient boosting* machine results in 83.92% of correct predictions. In detail, the boosting machine correctly predicts the 98.93% of negatives (i.e. not retweeted tweets) and only the 0,74% of the positives (i.e. retweeted tweets). By applying an extension of the algorithm, *xtreme gradient boosting* (Chen & He, 2020; Qian et al., 2020) we obtained an overall performance of 83.58% of correct predictions including the 96.93% of negatives (i.e. not retweeted tweets) and the 9.63% of the positives (i.e. retweeted tweets). Meanwhile, the traditional logit resulted in 83.24% of correct predictions. In detail, the logit models correctly predicts the 97.32% of negatives (i.e. not retweeted tweets) and only the 5.19% of the positives (i.e. retweeted tweets).

Overall, the traditional logit approach and the machine learning approach provide similar performances. However, the boosting machine displays more accurate ability to match the patterns of textual and non-textual cues in order to predict whether or not a tweet will be retweeted. Moreover, the ML approach is able to document the relative importance of the individual cue effects on retweets, offering a more nuanced explanation of the drivers of a Twitter user response to a tweet.

## Conclusion

This study aimed at investigating the textual and non-textual cues of tweets that affect their virality, focusing on the misinformation content during the COVID-19 pandemic. In the wake of the pandemic, indeed, social media platforms are playing a crucial role in the quick spreading of misinformation. Even though marketing literature is surprisingly scant on this topic, Twitter user-generated textual data provides unprecedented possibilities to analyse how individuals use language to communicate misinformation on social media. By analyzing a panel of 4923 tweets containing the hashtags #5G and #Huawei during the first week of May 2020, when several countries were still adopting lockdown measures, we determine whether or not a tweet is retweeted and, if so, how much it is retweeted. Overall, we found different effects of the textual and non-textual cues on the retweeting of a tweet and on its ability to accumulate retweets. In particular, the misinformation included in a tweet plays an interesting role in spreading the tweet through the network. We also applied a machine learning approach to investigate the pattern of textual and non-textual characteristics of a tweet in determining the Twitter users’ response in terms of retweeting behavior. We found this approach superior in reducing false positives and false negatives when compared to traditional logit predictions. More importantly, the relative influence of the cues suggests that Twitter users actually read a tweet but not necessarily they understand or critically evaluate it before deciding to share it on the platform. This supports the role of confirmation bias in affecting individuals’ susceptibility to misinformation as the tendency to propagate contents through social media largely depends on other textual cues than the veracity of contents.

The theoretical contributions of this paper are twofold: first, we contribute to the stream of literature regarding fake news from a marketing perspective which is still at a nascent stage (e.g. Di Domenico & Visentin, 2020; Di Domenico et al. 2021; Visentin et al. 2019). In particular, we provide evidence about the importance of textual cues in affecting retweeting behavior in the context of fake news and misinformation. Importantly, our findings suggest that these cues are not only related to the content of the tweet but also to the style used. Second, we contribute to the stream of literature that aims to analyze the features of tweets affecting virality (e.g. Berger et al., 2020), answering recent calls about the importance of providing models using language analysis and machine learning techniques (Valsesia et al., 2020).

From a managerial perspective, this study suggests that companies should continuously monitor tweets which are going to become viral in order to avoid the spread of misinformation not only inside the *echo-chamber*s but also outside them to avoid negative impact on the company’s reputation. Indeed, social media monitoring tools provide unprecedented possibilities to gather continuous minute-by-minute real-time data (Branthwaite & Patterson, 2011) that allow the tracking of the rapid changes of consumers’ sentiment over time (Zhang et al., 2011). As fake news and the other forms of misinformation targeting brands are recognized as reputational threats (Jahng, 2021), being timely in setting up a response strategy to misinformation is of paramount importance for brands (Chen & Cheng, 2019; Vafeiadis et al., 2019). In particular, the analysis of the linguistic patterns of misinformation could provide managers insights about what type of response could be more appropriate in recovering consumers’ trust, whether defensive (Vafeiadis et al., 2019; van der Meer & Jin, 2020) or accommodative (Coombs, 2015; Jahng, 2021). Moreover, having a deep comprehension of social media discussions might help brand managers exploit online communities’ brand attachment in defending the company’s reputation after a fake news attack, as suggested by the Nutella palm oil case (Cova & D’Antone, 2016). Furthermore, social media platforms are increasingly stepping up efforts to combat misinformation by strengthening their policies, in particular nowadays as the COVID-19 vaccines are introduced worldwide. This study suggests that in order to detect misinformation, social media platforms should not only focus on the content (i.e. misinformation) but also on how it is conveyed.

As a final note, in our analysis, we focused on a well-known company (i.e. Huawei) and a specific technology (i.e. 5G). However, misinformation is related also to other domains and during the pandemic, we registered different fake news and conspiracy theories targeting the pharmaceutical industry.In particular, a surge of misinformation has targeted vaccines, with anti-vax movements dramatically increasing their reach and threatening the uptake of immunization programs (Burgess et al., 2021). These conspiracies are nothing new, but the pandemic has created some conditions that pushed more and more individuals to embrace conspiracist thinking. Above all, the COVID-19 pandemic has further marginalised already oppressed minorities, including ethnic groups and people with disabilities (Johns et al., 2020) who are more likely to turn into conspiracies blaming powerful others for their disadvantaged living conditions (Douglas et al., 2019). As a consequence, individuals’ mistrust towards governments and health authorities has increased (Mylan & Hardman, 2021), making the pharmaceutical industry an easy target for conspiracy theories (Burgess et al., 2021).

Yet, Twitter users displayed a surprisingly poor brand awareness in the pharma industry as they were unable to mention individual brands, spreading tweets against *big pharma* as a non-specific entity. This might suggest companies monitor their brand awareness also on social media platforms and investigate how users mention them as the marketing discipline will not be likely the same after COVID-19 (Hoekstra & Leeflang, 2020). Future research might further investigate this topic and apply our analyses in other industries.
